# The complete chloroplast genome sequence of the medicinal plant *Nicandra physalodes* (Linn.) Gaertn. (Solanaceae)

**DOI:** 10.1080/23802359.2019.1666674

**Published:** 2019-09-18

**Authors:** Qi Chen, Dequan Zhang

**Affiliations:** a College of Pharmacy and Chemistry, Dali University, Dali, China;; b Institute of Materia Medica, Dali University, Dali, China

**Keywords:** *Nicandra physalodes*, chloroplast, Illumina sequencing, phylogeny

## Abstract

*Nicandra physalodes* is an alien plant in southern China, possessing important medicinal values. In this study, we sequenced complete chloroplast (cp) genome sequence of *N. physalodes* and investigated its phylogenetic relationship in the family Solanaceae. The total length of the chloroplast genome was 156,729 bp, with 37.8% overall GC content and exhibited typical quadripartite structure, a pair of IRs (inverted repeats) of 25,529 bp was separated by a small single copy (SSC) region of 18,560 bp and a large single copy (LSC) region of 87,111 bp. The cp genome contained 113 genes, including 79 protein-coding genes, 30 tRNA genes, and four rRNA genes. The phylogenetic analysis indicated *N. physalodes* was closely related to *Datura stramonium*.

*Nicandra* Adans. is a small genus in the Solanaceae, which only includes three species, namely *N. john-tyleriana*, *N. yacheriana*, and *N. physalodes* (Horton 1979). Among these species, *N. physalodes* is native to regions from Peru to northern Argentina (Hunznker 1979), then it was introduced to China as medicinal plant and now the species is widely found in tropical and subtropical areas of southern China (Zhang et al. 2000). In most regions, it has been used as folk medicine for sedative, expectorant, fever relieving, and detoxification (Editorial Board of National Herbal Compendium 1975) and its seed could be utilized to extract edible pectin to make jelly (Chen et al. 2017). However, for such a medicinal plant, most of the studies focused on its chemical compositions (Yi et al. 2012; Yu et al. 2017), but almost no genomic study was performed. Here, we reported the chloroplast genome sequence of *N. physalodes* and revealed its phylogenetic relationships with other genus in the Solanaceae.

Fresh leaf materials of *N. physalodes* were sampled from Yangbi County, Yunnan, China (N25.63°, E100.02°); meanwhile, a voucher specimen (No. ZDQ040) was collected and deposited at Dali University. Total genomic DNA was extracted using the improved CTAB method (Yang et al. 2014) and sequenced with Illumina Hiseq 2500 (Novogene, Tianjing, China) platform. The raw data were filtered using Trimmomatic v.0.32 with default settings (Bolger et al. 2014). Then, paired-end reads of clean data were assembled into circular contigs using GetOrganelle.py (Jin et al. 2018). Finally, the cpDNA was annotated using the Dual Organellar Genome Annotator (DOGMA; http://dogma.ccbb.utexas.edu/) and tRNAscan-SE (Lowe and Chan 2016).

The annotated chloroplast genome was submitted to the GenBank under the accession number MN165114. The total length of the chloroplast genome was 156,729 bp, with 37.8% overall GC content. With typical quadripartite structure, a pair of IRs (inverted repeats) of 25,529 bp was separated by a small single copy (SSC) region of 18,560 bp and a large single copy (LSC) region of 87,111 bp. The cp genome contained 113 genes, including 79 protein-coding genes, 30 tRNA genes, and four rRNA genes. Among these genes, 17 genes were duplicated in the inverted repeat regions, 16 genes, and six tRNA genes contain one intron, whereas two genes (*ycf3* and *clpP*) had two introns.

A total of 28 cp genome sequences of Solanaceae species were downloaded from the NCBI database used for phylogenetic analysis. The jModelTest v.2.1.7 (Darriba et al. 2012) was used to determine the best-fitting model for the chloroplast genomes. Then, Bayesian inference (BI) was performed using MrBayes v.3.2.6 (Ronquist et al. 2012) with *Scrophularia dentata* (No. NC_036942) as outgroup. The results showed that *N. physalodes* was closely related to *Datura stramonium* (Figure 1). The complete chloroplast genome of *N. physalodes* would lay a solid foundation for further phylogenetic studies.

**Figure 1.**
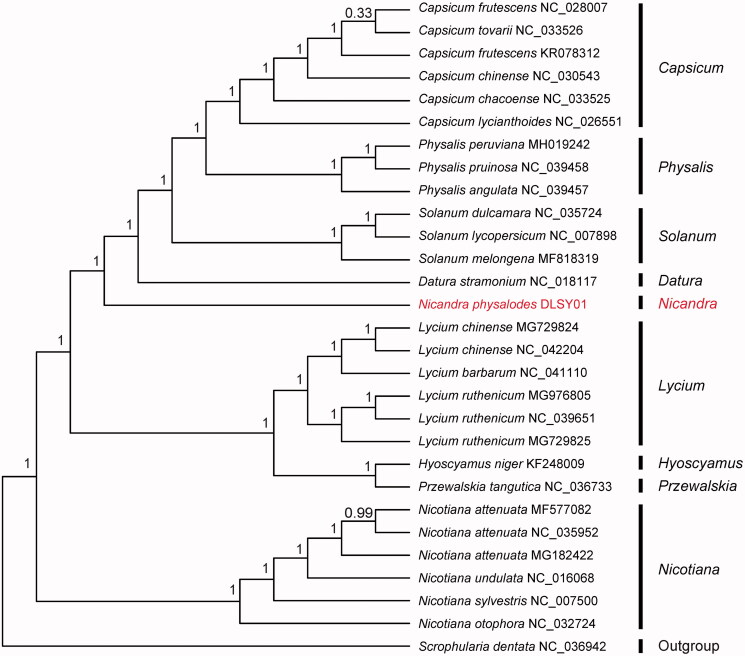
Phylogenetic tree of *N. physalodes* and related species in Solanaceae based on complete chloroplast genomes (*Scrophularia dentata* as outgroup).
